# Is planned adaptation to heat reducing heat-related mortality and illness? A systematic review

**DOI:** 10.1186/1471-2458-14-1112

**Published:** 2014-10-28

**Authors:** Melanie Boeckmann, Ines Rohn

**Affiliations:** Department Prevention and Evaluation, Leibniz Institute for Prevention Research and Epidemiology – BIPS, Achterstr. 30, 28359 Bremen, Germany; Center for Social Policy Research, University of Bremen, Mary-Somerville-Str. 5, 28359 Bremen, Germany; Medical University Hannover, Carl-Neuberg-Str 1, 30625 Hannover, Germany

**Keywords:** Heat, Climate change, Effectiveness, Systematic review, Cardiovascular disease, Respiratory disease

## Abstract

**Background:**

Extreme heat is an important public health risk. Climate change will likely increase the temperatures humans are exposed to through exacerbated heat wave intensity and frequency, possibly increasing health risks from heat. To prevent adverse effects on human health, heat prevention plans and climate change adaptation strategies are being implemented. But are these measures effectively reducing heat-related mortality and morbidity? This study assesses the evidence base in 2014.

**Methods:**

We conducted a systematic review of peer-reviewed published literature. We applied a combined search strategy of automated search and journal content search using the electronic databases PubMed, Web of Knowledge, Biological Abstracts, CAB Abstracts and ProQuest Dissertation & Theses A&I. Quality appraisal was conducted using CASP checklists, and we identified recurrent themes in studies with content analysis methodology. We conducted sub-group analyses for two types of studies: survey and interview research on behavioral change and perception, and observational studies with regression.

**Results:**

30 articles were included in the review. The majority of studies (n = 17) assessed mortality or morbidity reductions with regression analysis. Overall, the assessments report a reduction of adverse effects during extreme heat in places where preventive measures have been implemented. Population perception and behavior change were assessed in five studies, none of which had carried out a pre-test. Two themes emerged from the review: methodological challenges are a major hindrance to rigorous evaluation, and what counts as proof of an effective reduction in adverse health outcomes is disputed.

**Conclusions:**

Attributing health outcomes to heat adaptation remains a challenge. Recent study designs are less rigorous due to difficulties assigning the counterfactual. While sensitivity to heat is decreasing, the examined studies provide inconclusive evidence on individual planned adaptation measures.

**Electronic supplementary material:**

The online version of this article (doi:10.1186/1471-2458-14-1112) contains supplementary material, which is available to authorized users.

## Background

Extreme heat is a public health risk [1–3]. In 2013, 58.729 heat stroke diagnoses have been recorded for Japan [4], for example, and the United States Centers for Disease Control report an annual 659 cases (on average) of heat-related deaths between 1999 and 2009 [5]. These numbers are likely underestimated: as the physical effects of heat primarily exacerbate underlying conditions, diagnoses of death as heat-related are of varied quality [6]. Data availability on heat stroke incidence also depends on whether an emergency room or ambulance call occurs, as well as on active collection of such data. Heat increases the risk of dying of preexisting cardiovascular disease [6]; and heat stroke may lead to multiple organ failure [6–8]. Heat-related morbidity and mortality are preventable. Older persons, people taking medications that impair thermoregulation [6], very young children, socially isolated elderly, and people physically active outdoors during very hot periods have been identified as particularly at risk [9–12]. It has been argued that populations residing in urban centers are more vulnerable to heat events due to the urban heat island effect and higher population density [13–17]. In recent years additional concerns have arisen about a contribution of global warming to an increased frequency of extreme temperature events [18, 19]. “Business as usual” climate change scenarios estimate that the incidence of heat events is likely to increase in the near future [20, 21]. As a result, it has been suggested that future health risks from heat might increase [22–24]. In 2012, extreme temperature events classified as disasters by the WHO Collaborating Centre for Research on the Epidemiology of Disasters - CRED occurred 51 times worldwide, giving climatological disasters (temperature events, droughts and wildfires) an overall share of 23.8% of all 2012 disasters [25]. Recent severe heat waves occurred in Europe and Russia in 2003, 2006 and 2010, in the United States in 2012, in Australia in 2009 and 2013, and in Japan in 2010 and 2013, among others [26, 27]. Beyond these extreme cases, smaller scale heat waves occur frequently and pose risks to human health. Heat impacts on humans can be measured through thermal indices [28]. Various methods to calculate a heat index exist, and without adherence to a standard, comparability between measurements and studies is challenging [29, 30].

With a changing climate, populations of large cities in temperate regions, subtropical or tropical climates have all been characterized as vulnerable to heat [3, 31, 32]. Further measures may be needed to continually protect human health from adverse effects of heat on all continents. Adaptation to climate change has been defined as a “process of adjustment to actual or expected climate and its effects, in order to moderate harm or exploit beneficial opportunities” [33]. In this study, we are particularly interested in intentional, planned adaptation.

While we have conflicting information on risk perception of heat among populations [11, 34, 35], older persons have been characterized as especially susceptible to ill effects of heat [36–38]. Heat warning systems have been introduced as a prevention measure [39–41]. These usually combine information from weather stations based on a cutoff system with more or less targeted communication campaigns. Such heat warning systems can now be found across the planet, usually at city level [42].

Despite increased interest in climate change and its impacts, and a large number of heat prevention plans in place in higher-income countries to protect human health [39, 43], we have hardly any conclusive evidence on the effects of said adaptation measures [44]. Is climate change adaptation to heat reducing heat stroke incidence and heat-related mortality? This study uses a systematic review design in an attempt to answer this question.

## Methods

We conducted a systematic literature review of peer-reviewed published literature. The PRISMA checklist, research protocol and the data extraction sheet can be found in the supplementary material (Additional files 1, 2 and 3). The scope of our review was as follows:

**Population:** urban populations of all ages, sexes and ethnic groups.

**Intervention:** Heat adaptation measures conducted in an urban area.

Because heat adaptation aims at preventing adverse health effects, we use the terms heat adaptation and heat prevention interchangeably in this review.

**Comparison:** none (no adaptation).

**Outcomes:** impacts on heat-related morbidity and mortality.

**Context:** International large urban centers^a^.

The following outcomes were of interest:

- Impacts measured as reduction in excess heat stroke incidence, hospitalization for heat-related illness, and cases of cardiovascular, respiratory and all-cause mortality in extreme heat periods as compared to previous heat periods.
- Effectiveness measured

1. as reduction in excess heat stroke incidence, hospitalization for heat-related illness and cases of cardiovascular, respiratory and all-cause mortality, for which we accepted the proxy indicator of health services use (emergency medical care at facility or on ambulance; hospital release diagnosis or physician’s diagnosis) for heat stroke,
2. as heat island exposure reduction signaled through changes in urban planning or taking up of heat warning systems.

### Search strategy

We applied a combined search strategy of automated search and hand search of journals. Two researchers independently searched the electronic databases PubMed, Web of Knowledge, Biological Abstracts, CAB Abstracts and ProQuest Dissertation & Theses A&I.

We applied combinations of the search terms climat*, heat, adapt*, compounds of climate change, adaptation, adapting, heat wave, extreme heat, heat island combined with evaluat*, effect* and exposure in the automated searches^b^.

Search strings had been pre-tested during a mapping review.

Additionally, both researchers manually searched the journals Climatic Change and International Journal of Climate Change Strategies and Management to increase our chances of finding articles that focus on evaluating adaptation strategies from a management or urban planning perspective.

Ancillary search procedures included checking the reference lists of identified primary studies as well as asking three leading international researchers for suggestions and works in progress.

### Selection criteria

The following inclusion and exclusion criteria were applied:

#### Inclusion

Must include adaptation specifically for heat. All languages as long as an English abstract is available. Only reviews and original research articles as well as books or published national and international reports (defined as having an ISBN number). Must include at least one human health outcome, or health-related behavior changes. Must contain an evaluation or assessment. All publication years included.

#### Exclusion

No English abstract available. Comments, editorials, correspondences and letters are excluded. Mitigation rather than adaptation focus of the article. Focus too limited: only a description of heat adaptation planned or implemented without assessment of effects. No evaluation of human health impacts.

Two researchers independently selected relevant articles from the searches with the same search terms as well as through cross-checking reference lists. One researcher contacted leading experts for input on work-in-progress and further studies to be included via email.

Any disagreement between the two researchers was resolved and evaluated by a third member of the research team.

### Study quality assessment

For study quality assessment, the NHS Critical Appraisal Skills Program (CASP) [45] checklists were used according to each study type. CASP also provides a checklist for quality appraisal of qualitative studies. Although specific tools for each study type prohibit a general comparison across study types, Katrak et al. [46] have previously criticized generic assessment tools for being too general. In addition, our review aimed at being comprehensive and therefore intentionally included a vast range of studies. Any attempt to assess these with a generic tool was unfit for representing their diversity. The CASP checklists were aimed at answering general guiding questions also provided by Booth et al. [47]:

1. Validity: Do the results of a study fit with other available evidence? How are confounding and bias handled?
2. Reliability: What are the results and how much might they be owed to chance?
3. Applicability: Can we generalize the results? How strong are recommendations for practice based on these study results?

For the specific questions, see Additional file 4.

To reduce the risk of subjective quality judgment, we decided not to exclude nor weigh studies based on quality rating or scales. While study quality assessment is important to judge the overall evidence base for adaptation effectiveness, the usefulness of excluding studies based on quality has been contested [47, 48].

### Study synthesis

Due to the heterogeneity and varied designs of studies and reports, no overall quantitative meta-analysis could be performed. Instead, we applied narrative synthesis.

We conducted two subgroup analyses of survey studies and observational studies as these were the two most common study types.

## Results

**Figure 1:**
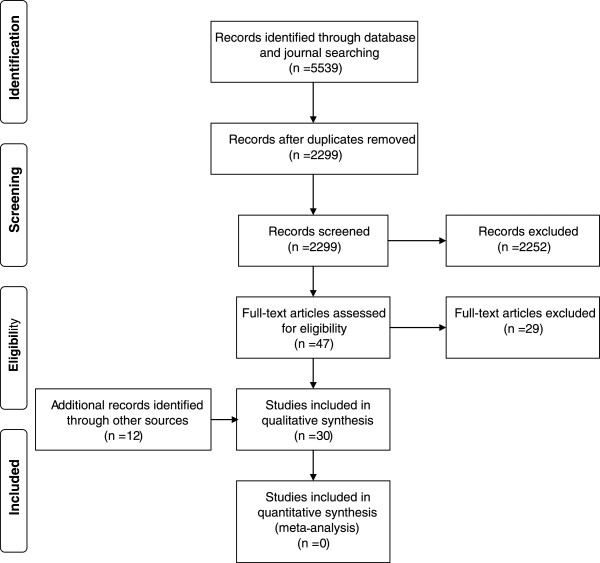
**PRISMA flowchart of study selection process.**

The database search led to 5539 results, 2299 after removal of duplicates. After title and abstract screening 2252 articles were excluded because they did not concern human health or did not contain an evaluation. 47 articles were assessed as full texts. We excluded 29 articles after reading the full texts because no evaluation according to our criteria was described. Through additional sources such as reference lists we identified 12 studies. All in all, 30 articles were included in the review, as shown in the PRISMA flowchart (Figure 1).

### Study characteristics

**Figure 2:**
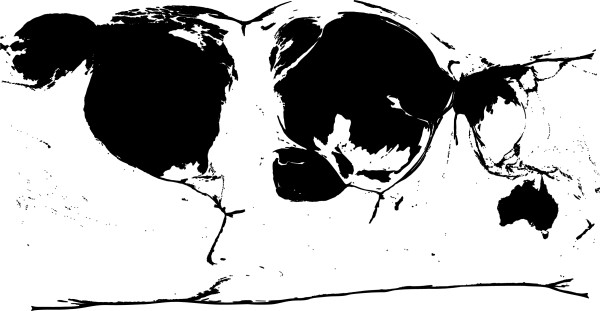
**Continents of study origin.** Distorted cartogram of continents of study origin, weighted by number of studies per country. Countries with higher number of publications are larger in the cartogram.

Of the 30 articles, 12 were studies conducted in European countries [35, 49–59], 10 studies were from the United States, one of which included a Canadian study city [34, 60–68], two from East Asian countries [69, 70], one from Canada [71], and one from Australia [72]. The systematic reviews were not restricted to any continent [73–76]. Figure 2 shows the imbalance of country of origin for the publications in a distorted cartogram [77]: more studies were published in higher-income, Western countries versus lower-income countries. Countries with a higher output are represented as larger in the cartogram (Figure 2). We did not identify any studies from Africa, Southeast Asia or Central and South America. The Pacific Region was also underrepresented.

Time of publication ranged from 1992 to 2013 (median = 2008). Regarding study population, about one-third of the studies focused on older persons (n = 11) [35, 49, 50, 52, 57–59, 61, 66, 67, 69]. However, definitions of an older person differed and ranged from inclusion of over 64 to 75 and beyond. The remaining studies included all adults aged 18 years and older.

Half of the identified studies were observational studies with regression as main analysis method (n = 16) [49, 51–53, 55, 56, 58–60, 63–65, 67–70], followed by survey research (n = 6) [34, 54, 62, 66, 71, 72]. We identified two qualitative interview studies [35, 50], one randomized controlled trial (RCT) [57], one economic analysis [61], and four systematic reviews [73–76]. Additional file 5 describes characteristics of the studies included in the review.

### Heat adaptation

**Figure 3:**
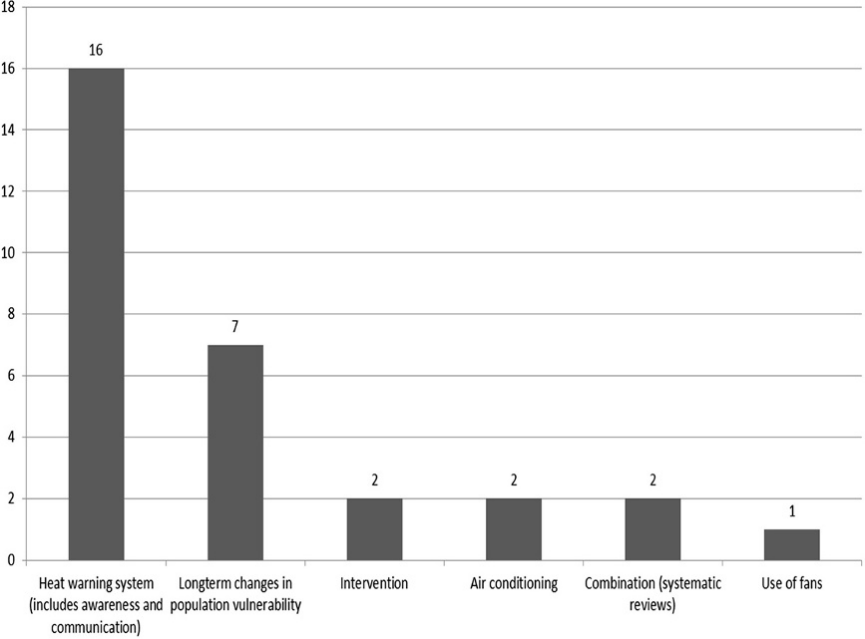
**Type of adaptation in studies included in review.** Adaptation measures discussed in the individual studies.

Adaptation options to heat assessed in the included studies ranged from heat warning campaign communication [35, 49, 50], use of fans [75], and active surveillance programs [57] to biological acclimatization over decades [55, 56] (Figure 3).

Main outcomes were mortality rate trends over several years, mortality rates pre- and post-intervention, and changes in awareness or behavior over time. Due to limited comparability of the studies, specific study results will be discussed under subgroup analysis for regression analysis and perception survey results (Tables 1 and 2).

**Table 1:** **Results of the regression analysis studies and RCT**

***Reference***	***Type of evaluation***	***Results***
Rogot et al. 1992 [65]	Comparing mortality during heat in people with air conditioned homes to those with no air conditioning	Central air condition compared to no air condition: OR below 1 for all groups, significant (p = 0.03 Mantel-Haenszel). **Room air condition compared to no air condition: OR 0.96 for total group, p = 0.71). RR for central air condition vs. no air condition 0.58 for total group,** RR for room air condition to no air condition 0.41 for total group
Smoyer 1998 [67]	Comparing mortality rates of 1980 and 1995	The average elderly **mortality rate on heat wave days** went down **from 2.36 (SD 1.20) to 1.65 (SD 0.52)**, the average elderly mortality rate on non- heat days went down from 1.56 (SD 0.45) to 1.46 (SD 0.55)
Palecki et al. 2001 [64]	Comparing excess deaths in 1995 and 1999	**Mortality rates in Chicago and St Louis both 1.4 per 100.000 in 1999,** if not using core cities but counties. In 1995, 700 died in Chicago and 27 in St Louis
Weisskopf et al. 2002 [68]	Changes in population vulnerability	Model 1: predicted mortality rate of 1.80 per heat-index degree above 80 °F. 42.3 expected deaths, actual deaths in 1999 were 10. Model 2: **RR** for heat-related death in 1999: **0.17-0.24, RR** for emergency medical services in 1999 **0.32-0.46**
Davis et al. 2003 [60]	Comparing temperature mortality relationship from 1964 to 1998	The threshold for 1960s-1970s is no longer connected to an increased mortality in the 1980s in Northeastern cities, and in the 1990s 10 show no elevated mortality above threshold and of the remaining 18 cities 12 show a decline in mortality rate. **Six cities** remain with an **increased mortality rate** above the threshold: Atlanta, Buffalo, Dallas, Denver, Seattle, San Francisco
Delaroziere and Sanmarco 2004 [52]	Comparing mortality before and after implementation of warning system	Mean **index of daily excess mortality** has dropped from 3.27 in the years 1986 to 1982, **down to 1.32** in the years 1984–1997, p = 0.008)
Marinacci et al. 2009 [57]	Comparing no. of hospitalizations and deaths in summer 2004, RCT	Males: in intervention group Odds to be emergency hospitalized: **OR 0.33, 95% CI: 0.11; 0.96.** Females: in intervention group odds to be hospitalized overall: **OR 0.96, 95% CI: 0.93; 0.98**
Tan et al. 2007 [70]	Comparing daily excess mortality in 1998 and 2003.	**Correlation coefficient** between daily deaths and weather and air pollution parameters: death and time of heat wave: **0.34 in 1998 and 0.41 in 2003,** Tmax in 1998 0.51 to 0.62 in 2003. Heat related deaths in 1998: 358 (absolutes), 253 in 2003 (absolutes)
De’Donato et al. 2008 [51]	Daily excess mortality before (reference period) and after implementation of heat warning system	J-shape temperature-mortality curve in all cities. In Milan and Rome in 2007 there was a weaker association between high temps and mortality. In Bari and Catania there was a greater impact of high temp on mortality in 2007 (all compared to 2003). In 2007 excess mortality occurred during three heat waves, with **impacts on mortality of +10-41% in the center and 11-56% in the South**
Fouillet et al. 2008 [53]	Comparing excess daily mortality in 2003 to 2006	During summers 2004 and 2005, observed no. of deaths was 2-8% lower than predicted no. of deaths. In 2006 2065 excess deaths occurred, predicted for that temperature were 6452 excess deaths, **4400 fewer deaths than predicted**
Kysely and Kriz 2008 [55]	Comparing excess mortality in the 1990s and 2003	Excess daily mortality in 1990s: 98 deaths in 1992, 113 deaths in 1994; 50 deaths in 2003. Aggregated: 1992 718 excess deaths, in 1994 919 excess deaths, in 2003 **236 excess deaths**
Bargagli et al. 2009 [49]	Mortality rate among patients with active surveillance and those without = comparison of mortality rate with and without intervention	**Excess mortality on heat days vs. non-heat days in controls: RR 1.20, 95% CI: 1.14-1.27**; excess mortality on heat days vs. non-heat days in **intervention patients: RR 0.95, 95% CI: 0.65-1.34**
Chau et al. 2009 [69]	Comparing associations between hot weather warning and mortality rates from ischemic heart disease and stroke from 1997 to 2005.	Absence of warning system was associated with an increase of **1.23 deaths** from IHD (95% CI 0.32; 2.14), an increase of **0.97 deaths** from stroke (95% CI: 0.02; 1.92) per day
Ostro et al. 2010 [63]	Comparing hospitalization among those with air conditioning to those without	Reduction in excess risk of hospitalization with 10% increase in A/C ownership: respiratory disease: **relative reduction 19.9% (95% CI 0.7;39.), CVD relative reduction: 49.1% (95% CI 19.9;78.3), heat stroke relative reduction 4.0% (95% CI 1.9;6.0)**
Kysely and Plavcova 2012 [78]	Comparing temperature mortality relationship from 1986 to 2009	Significant **trends in deviation of mortality on lag days from 1986 to 2009: all ages D + 1 -0.61, D + 2 -0.55; 70- years: D + 1 -0.66; 70+ years: D + 2 -0.66.** Relative deviations of mortality declined by 0.4% to 0.5% in all age groups until 2009. **Overall decline of mortality by 10% for all groups**
Morabito et al. 2012 [58]	Comparing mortality before and after implementation of warning system	Odds Ratios for mortality by age group pre- and post-2003: only significant in 75 years+, OR for average apparent temperature before 2003 **1.18 (CI 1.10-1.26),** 2004 to 2005: **1.24 (CI 1.14-1.35)**, 2006–2007: **1.20 (CI 1.09-1.31).** Also significant for maximum temperature
Schifano et al. 2012 [59]	Comparing daily mortality in 1998–2002 (before) and from 2006 to 2010 (after) implementation of prevention program	**Weaker relationships between heat and mortality in all 16 cities post-intervention.** Percentage change in mortality per 3°C increase in max apparent temperature MAT (pooled results): for 0 to 3% increase of 3°C increase: 1998–2002: 5.65%, for 2006 to 2010: 5.65%; 3 to 6% MAT increase: in 1998–2002 6.72% change, in 2006 to 2010: 7.79% change. Largest results: **12 to 15% MAT increase, 41.76% change from 1998–2002; 5.65% change from 2006 to 2010**

Main results are in bold.

**Table 2:** **Results of reviews, survey studies qualitative interview studies and economic analysis**

***Reference***	***Type of evaluation***	***Methods***	***Results***
Mattern et al. 2000 [62]	Case-only survey	Standardized questionnaire	34 respondents. At pretest 67% of respondents knew whom to contact during heat for assistance, post-intervention 94% knew whom to contact. 6% knew about the City of Philadelphia hotline at pretest, 29% at post-test. **76% monitored temperature daily,** 21% monitored temperature during hot days
Ebi et al. 2004 [61]	Economic cost-effectiveness evaluation	Multiple linear regression, estimation of lives saved, estimation of benefits	**2.6 lives saved** on average for each warning day plus three day lag (not significant). Estimated value of $6.12mill. per life = **$468 mill. saved with 117 lives saved over 3 years.** Costs for system $210.000
Kishonti et al. 2006 [54]	State of knowledge on heat, the warning system, protective behavior	Quantitative telephone survey	Sample size 2500. Awareness of heat: persons between 30 and 59 years of age mentioned at least two health impacts of heat. 27% of respondents saw hypertension as risk, 11% heat stroke, 22% CVD. **25% of interviewees had seen the communication campaign**, of whom 78% saw it on TV, 57% in the newspaper and 41% on the street. **59% of respondents had heard of heat alarm**
Bouchama et al. 2007 [74]	Systematic review and meta-analysis on risk and protective factors for heat-related deaths	Systematic review and meta-analysis	Protective factors: home air condition **(OR 0.23 95% CI 0.1-0.6)**, visiting cool environments **(OR 0.34 95% CI 0.2-0.5)**, increased social contact **(OR 0.40 95% CI 0.2-0.8)**, taking extra showers **(OR 0.32, 95% CI 01.-1.1)**, use of fans **(OR 0.60 95% CI 0.4-1.1)**
Kalkstein and Sheridan 2007 [34]	State of knowledge on heat, the warning system, protective behavior	Quantitative survey	201 respondents, 14 of age 65+. 90.2% of females knew about the heat warning system, 75.3% of males knew about the system. 25% felt heat was dangerous. Of those aware of heat warnings, **49.7% altered behavior**, 47.3% did not
Sheridan 2007 [66]	State of knowledge on heat, protective behavior, available cooling systems in the house	Quantitative telephone survey	908 respondents across all cities. In the four cities, **most people learned about heat warnings on television** (Dayton: 89%, Philadelphia: 84%, Phoenix: 92%, Toronto: 64%). **46% of respondents altered their behavior during heat**, varying significantly across cities (p = 0.003). Use of air conditioning self-restricted due to concerns about costs
Abrahamson et al. 2009 [35]	State of knowledge on heat-related health risks and protective behavior	Semi-structured interviews with topic guide, 1 data collection wave summer of 2007	73 respondents, mean age 81 years (range 72–90) in London; mean age 80 (range 75 to 94) in Norwich. Themes identified: perception of vulnerability to heat; behavior change during heat; knowledge of protection measures; perception of usefulness of heat wave plan. **No consensus on usefulness of heat wave plan components**. Most respondents adjust their behavior during heat. Few respondents perceived of themselves at risk
Kosatsky et al. 2009 [71]	State of knowledge on heat, protective behavior	Quantitative, questionnaire based face-to-face interviews	238 respondents. 86% know about risks of high night time temperature, 94% know about health risks for lung and heart disease patients. 80% listen to weather forecasts, mid-summer **93% had heard a heat advisory**. 71% use a fan, 87% do less strenuous activities in heat. 73% have air condition at home, those with air condition reported more additional behavior changes than those without
Bassil and Cole 2010 [73]	Systematic review of all study types	Systematic review and expert elicitation	Narrative results: **most studies evaluate heat warning systems, awareness and perception.** If effects measured then often as regression analysis. Methodological challenges
Oakman et al. 2010 [72]	State of knowledge on heat, heat warnings, protective behavior	Quantitative telephone survey	328 interviews, **63% knew of health warnings**: of these 74% saw it on TV, 42% on radio, 15% in newspapers. 96.1% of respondents used air condition in hot weather, 94% drank water, 90% stayed indoors
Bittner and Stößel 2012 [50]	State of knowledge on heat, protective behavior, heat warnings	Questionnaire-based interviews, qualitative analysis with framework approach	20 respondents. Themes: vulnerability, changes in daily routine, sources of information, content of advice received, activity level and health status. **Individual vulnerability not always perceived.** Controversial role of the GP. 19 respondents stated they changed behavior
Gupta et al. 2012 [75]	Systematic review of RCTs, and experimental designs with controls	Systematic review according to Cochrane guidelines	**No studies with rigorous experimental designs found**
Toloo et al. 2013 [44]	Systematic review of any heat warning evaluation	Systematic review of databases	Six articles asserted that post-intervention expected deaths were reduced. **High study heterogeneity.** One economic assessment. Eight studies assessed awareness, including one qualitative study

Main results are in bold.

### Quality appraisal

We used the CASP checklists [45] to assess study quality. As expected from scoping literature searches, studies included in the review were highly heterogeneous in research question and design. We used the CASP checklists for RCT (n = 1) [57], systematic reviews (n = 4) [73–76], qualitative studies, also used for survey research (n = 7) [34, 35, 50, 54, 66, 71, 72], case–control studies including one survey-based case–control study (n = 15) [51–53, 55, 58–60, 62–64, 67–70, 78], economic analyses (n = 1) [61] and cohort studies (n = 2) [49, 65]. Results of the quality appraisal are presented in Additional file 4. Although we did not assign a quality score, we were able to see two main challenges for research design in the studies that may compromise quality: for survey and qualitative research on awareness changes, no baseline assessment was performed. For regression analyses, the definition of a control was not standardized.

### Subgroup analysis: articles comparing mortality and morbidity

**Figure 4:**
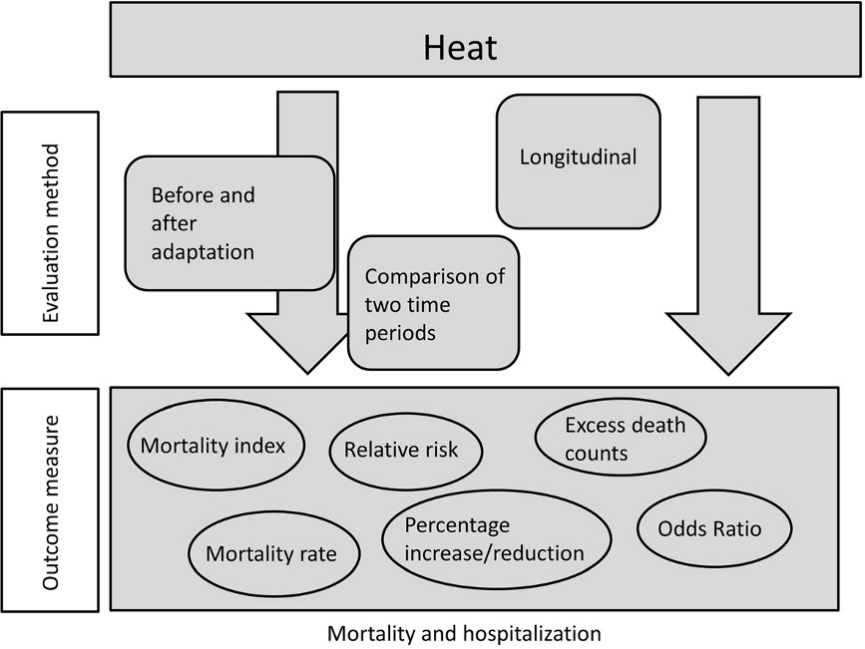
**Model of the variability in reported outcomes.**

The majority of articles (n = 17) compared mortality or morbidity, either over a period of several years or before and after implementation of a heat wave warning system. Study types in this assessment included one RCT [57], 14 case–control studies [51–53, 55, 58, 59, 63, 64, 67–70, 78, 79] and 2 cohort studies [49, 65]. However, the variety of outcomes reported prevented us from combining results in a meta-analysis (Figure 4).

Outcomes were reported as odds ratios, mortality rates, excess deaths, relative risk, increased percentage of mortality per centigrade temperature increase, or as a mortality index. Table 1 shows results of these studies. The studies were of high quality using standard epidemiological methods. Overall, the majority of assessments report a reduction of adverse effects during extreme heat. This applies both to longitudinal and short-term studies. For instance, Chau et al. [69] report an increase of 1.23 deaths from ischemic heart disease in Hong Kong where a heat warning system was absent between 1997 and 2005. For the cities in the United States, on the other hand, Davis et al. [60] find an increased heat-related mortality rate since 1964 for Atlanta, Buffalo, Dallas, Denver, Seattle and San Francisco. In Central Europe, Kysely and Plavcova [78] describe an overall decrease in mortality by 10% from 1986 to 2009. A common challenge for the studies is linking the decrease to specific adaptation measures: alternative hypotheses for the observed declines in sensitivity have not been tested.

### Subgroup analysis: perception and behavior change studies

The second largest group of study types was comprised of awareness and perception surveys and interviews. The articles all shared a common definition of awareness and behavior change. No pre-test was conducted in the survey and interview studies except for the study by Mattern et al. [62]. Results are presented in Table 2.

Most participants were informed of risks of extreme heat through media, television being the most common [34, 54, 66, 71, 72]. Individual adaptation behaviors were use of air condition, drinking water and avoiding strenuous activities [34, 35, 50, 54, 66, 71, 72]. Risk perception was discussed in the publications by Abrahamson et al. [35] and Bittner and Stößel [50]: both discovered that among their participants, older persons did not feel more at risk than younger populations. Concern about the costs of increased air condition use was mentioned by Sheridan [66]. We argue that due to the lack of pretests, the success of behavioral intervention advice cannot be estimated conclusively as it cannot be compared to knowledge and behavioral habits prior to the implementation of an adaptation measure.

## Discussion

The results of our review reveal difficulties in assessing adaptation effectiveness and are consistent with previous research. This suggests that issues of methodological rigor and what to measure when speaking about effectiveness of heat adaptation have not yet been resolved, despite increased interest in the matter.

Common themes in all studies were difficulties assessing adaptation effectiveness with standard epidemiological methods. This has been discussed particularly in the four systematic reviews. Specifically, the following issues in conducting rigorous studies to generate conclusive evidence of adaptation effects have been named:

- Differing heat wave impacts due to unstable intensity and frequency [76].
- Role of confounders such as socio-economic variables and long-term healthcare improvements [76].
- Short time frame between implementation of heat prevention and evaluation [73].
- Location-specific acclimatization [73].
- Simultaneous implementation of sub-interventions in a heat prevention plan [73].
- Data availability [76].

Gupta et al. [75] call for experimental study designs to assess the effectiveness of using fans during a heat wave as they were unable to resolve conflicting information from observational studies in their Cochrane review. In our included studies a call for more rigorous methods was the standard solution to the above mentioned issues, without specific recommendations on how to achieve this. When trying to judge whether the information we gathered through the review is sufficient proof that heat adaptation reduces heat-related mortality and illness, we struggle with the following problems posed by the available studies:

- Although older persons are generally included as a vulnerable group, age ranges differ and impede comparability.
- Lack of pre-tests in awareness studies. Participants’ knowledge of heat warning systems or healthy behaviors cannot clearly be attributed to the adaptation.
- Most of the observational studies did not examine alternative hypotheses for changes. Often authors mentioned a variety of reasons for changes, all of them with equal or unknown likeliness.

Why is conducting experimental research of adaptation to heat so difficult? For one, defining the counterfactual, i.e. what would have happened in the absence of the adaptation measure, is problematic, because usually an entire city or even country is exposed to the adaptation measure. Choosing a different city as control would require careful matching. This is difficult for many reasons: for example, intercity microclimate variability could bias results, and to assess effects the control city would need to be exposed to a heat event of similar magnitude and length. Unlike other public health interventions, researchers and practitioners cannot limit exposure; they can only mediate it.

Second, heat prevention can occur at structural level, or at individual level through behavior change. Ethical concerns could be raised if structural prevention or a warning system were only available to an intervention group in one city. For instance, control populations could not be prevented from accessing public green spaces.

Third, heat by itself is not a new phenomenon. Much of the heat-related health advice provided by risk communication campaigns is common sense information: to stay hydrated, for example, or to seek shade and cool places [80]. Physical discomfort during heat makes it likely that people have followed such advice before official warnings were even issued. This might not only suggest absence of the classic control group for behavior, it is also more difficult to compare knowledge pre- and post-information campaigns. In light of future population aging, potential improvements to adaptation effects lie with targeting those elderly people who do not feel at risk through awareness raising interventions despite these difficulties. The use of innovative materials and social norms approaches could be evaluated.

While we argue that concrete evidence for the effectiveness of specific planned adaptation measures is lacking, our results show a mostly unanimous decline in sensitivity to heat over longer time periods. Alternative hypotheses for the causes of this decline should be investigated. Proposed alternatives have included biological adaptation [81], improvements to healthcare systems [82], technological advancements [83], adjustments to the urban built environment [84], and social progress [84]. The role each of the alternatives plays in declining heat sensitivity is debated [78].

Aware of these shortcomings, recent research projects into methods specifically for adaptation assessments have been designed [85], results are not yet available.

We were surprised to be unable to identify articles assessing infrastructural measures such as greening, or supply of air conditioning, although we had specifically intended to include these. Our focus on human health and our health–related search terms may have prevented us from finding articles on urban planning effects. Connecting specific urban planning to public health assessments might be a challenging but interesting future research topic.

### Policy implications

With such little conclusive evidence of effectiveness, recommendations for future action need to be carefully considered. On the one hand, policymakers may feel a moral imperative to act regardless of the evidence base. On the other hand, negative health effects of the adaptation measures themselves should be avoided. Possible risks from adaptation include misinformation on protective behaviors leading to maladaptation, or increased allergic disease incidence through greening of urban spaces [86]. Using “low-regret” adaptation measures could be an interim solution until more suitable assessment methods have been developed. In climate change adaptation, low-regret options are generally all strategies that either offer more than one benefit or keep options for amendments open [87, 88]. Such options have been described as useful when uncertainties are large, as they do not rely on exact climate change projections [88, 89]. They yield a number of benefits for a system’s capacities to deal with climatic changes while only requiring moderate input, and are less likely to have negative effects [87, 88]. In practice, benefits will have to be weighed against opportunity costs and trade-offs [90].

Examples for popular low-regret options in heat adaptation might be urban greening and heat wave warnings [91]. However, creating such an inventory of low-regret measures does not actually solve the issue of whether adaptation works. A prominent voice in climate change and health research, Anthony McMichael, argued that a focus on traditional epidemiological assessments methods may not lead to increased knowledge as desired [92]. Instead, McMichael wrote, taking risks with new concepts, methods and interdisciplinary approaches to research are required [92].

### Limitations

In this review, we focused on peer-reviewed literature and excluded all unpublished or grey literature directing main attention towards database searches. This was justified by our specified interest in evidence of effectiveness as proven by rigorous scientific research, rather than in any evaluation possibly conducted by practitioners. A previous review from 2010 [73] stated that grey literature would be a more likely source of effectiveness information than peer-reviewed journal articles owing to the low number of evaluations conducted in research. Nonetheless, Bassil and Cole [73, 93] only found one unpublished study that contributed to the information on effects. As there is no legal imperative for policymakers in Europe to evaluate adaptation strategies, for example, few assessments are undertaken [94]. We aimed for comprehensiveness and therefore included non-health related databases to search for infrastructural evaluations. The final article selection, however, was entirely from academic health and medicine journals. This suggests that even if evaluation of green spaces or other infrastructural measures occur, these evaluations are less likely to consider co-effects on human health.

We identified no articles from Africa, Southeast Asia, the Pacific or Central and South America. This confirms previous findings on a dominance of high-income Western countries in adaptation research [95].

Nevertheless, we were able to identify 30 articles dealing with issues of evaluating heat adaptation, a large number in light of the novelty of adaptation and evaluation research. By our subgroup analysis approach, we contributed to knowledge on effectiveness as generated by two current adaptation evaluation standards: awareness surveys and mortality rate comparisons. Our review identifies major challenges to evaluation and proposes further research into the potential of adaptation measures for health protection from extreme heat.

## Conclusions

Our results show that rigorous evaluation of adaptation is rare and difficult to conduct. The potential health effects of adaptation can currently not be measured conclusively. Up to now, we find limited intersectoral efforts between public health agencies and climate change adaptation policy. Such efforts might contribute to a reduction in adverse health effects of heat. In addition, involvement of the health sector in adaptation design, implementation and evaluation might increase chances of successful adaptation.

Current knowledge does not prove effectiveness of planned adaptation, yet a decline in sensitivity to heat hints at important developments. Recent articles published after the search period for this review observe a similar decline over long time periods [96–98]. Whether biological adaptation, continuous improvements in healthcare, changes to the urban environment not declared “adaptation,” or a different unknown reason caused said decline is a matter of further interest. The seeming paradox between the observed decline in the examined studies and scholarly works referring to an expected increase in heat-related adverse health effects [99] needs to be assessed further as well. Low-regret adaptation options might be investigated while simultaneously increasing efforts to overcome methodological evaluation challenges with further research.

## Endnotes

^a^Originally we had planned to include only cities with more than 500,000 inhabitants. Due to the limited study availability, however, we decided to broaden this criterion to cities of any size.

^b^* = wildcard, all possible word endings included.

## Electronic supplementary material

Additional file 1: **PRISMA checklist.** (PDF 208 KB)

Additional file 2: **Review protocol.** The protocol for the systematic review. (PDF 334 KB)

Additional file 3: **Data extraction sheet.** (PDF 209 KB)

Additional file 4: **Quality appraisal results.** The results of the quality appraisal conducted with CASP checklists. (DOCX 24 KB)

Additional file 5: **Table of characteristics of studies included in review.** Studies are presented ordered by type and year of publication. (DOCX 21 KB)
