# Supplementary material for: Is planned adaptation to heat reducing heat-related mortality and illness? A systematic review
Source: BMC Public Health. 2014 Oct 28;14:1112. doi: 10.1186/1471-2458-14-1112 (PMC4219109; doi:10.1186/1471-2458-14-1112)
Supplement: Supplementary file 2 — Additional file 2: Review protocol. The protocol for the systematic review. (PDF 334 KB) [file 12889_2014_7199_MOESM2_ESM.pdf]

Is adaptation to heat reducing heat-related mortality and illness? A systematic review

Systematic Literature Review Protocol

1. **Change Record**

04.01.13:
-defined outcomes
-further defined quality appraisal process
-added to analysis procedure
-added study limitations
-defined operationalization
-clarified and focused research question
-added scope
-amended timeline

14.01.13:
-additional operationalization of "heat stroke incidence"
-clarification of "awareness" as an indicator
-clarification of how effectiveness is measured.

06.03.13
-amended timeline
-specified search terms

07.03.13:
-refined database selection

12.03.13
-finalized database selection

15.03.2013
-changed journal and additional database selection for manual search based on content

10.10.2013
-amended timeline

2. Background

Climate change and global warming will likely contribute to a higher frequency and

intensity of extreme temperature events (SREX 2012). Extreme heat and heat waves have led to high mortality in industrialized nations in recent years (Honda et al. 2010; Fouillet et al. 2008; Pascal et al. 2012). Cities are a high-risk environment for adverse health effects of heat because of dense population patterns, lack of green and open spaces as well as the urban heat island effect. Adaptation has been proposed as a strategy of preparing for changes in weather and climate that cannot be prevented by mitigation (IPCC 2007). However, it is unknown how effective adaptation to heat is. This review aims to identify these gaps in knowledge on adaptation effectiveness

The main research question addressed in this study is

Is heat adaptation in urban areas reducing heat stroke incidence and cardiovascular mortality?

Scope of the review: (PICOC statement)

Population: urban populations of all ages, sexes and ethnic groups.

Intervention: Heat adaptation measures conducted in an urban area.

Comparison: none (no adaptation)

Outcomes: impacts on heat stroke incidence and cardiovascular mortality. Impacts are measured as reduction in heat stroke incidence and cases of cardiovascular mortality in extreme heat periods.

Effectiveness is measured 1) medically as reduction in heat stroke incidence and cardiovascular mortality.

Regarding heat stroke incidence, the proxy indicator of health services use (emergency medical care at facility or on ambulance; hospital release diagnosis or physician's diagnosis) is accepted.

Effectiveness is measured 2) by heat island exposure reduction signaled through changes in these risk factors (exists/does not exist AND/OR number of documents/campaigns/shaded buildings/plans/warning systems prior to and after adaptation implementation):

- Heat-appropriate behavior education campaign (awareness-raising campaigns)
- green roofs,
- air conditioning,
- shaded buildings,
- early heat wave warning systems,
- risk communication strategies.

Context: International large urban centres (>500,000 inhabitants).

Adaptation recommendations are usually based on the knowledge about inevitable changes in climatic condition. They have not, however, been evaluated yet (Hosking & Campell-Lendrum 2012). We do not know if adaptation to heat can work and if so, how effective can it be? Thus a new study is needed to address the issue of evaluation and measures of effectiveness.

3. Search Strategy

The entire review will be guided by the PRISMA statement.

In this review, a combined search strategy of automated search and hand search will be applied. We will search the electronic databases **PubMed, Web of Knowledge, as well as Biological Abstracts (University of Hannover) CAB Abstracts (University of Hannover)** and **ProQuest Dissertation & Theses A&I (University of Hannover).**
The search engine GOOGLE scholar will be consulted as well to identify additional documents.
Search terms will be used in MeSH as well as in titles and abstracts. The following core search terms are used in the automated searches (*= wildcard, all possible word endings included): climat*, heat, adapt*, compounds of climate change, adaptation, adapting, heat wave, extreme heat, heat island combined with evaluat* , effect* and exposure. Search strings were already pre-tested during a mapping review.
The specific search strings are available in the appendix.

The following journals will be manually searched: **Global Environmental Change, Wiley Interdisciplinary Reviews: Climate Change, Nature, ~~Science~~, Climatic Changes, International Journal of Climate Change Strategies and Management.**

Ancillary search procedures are: checking the reference lists of identified primary studies as well as asking leading researchers for suggestions and works in progress.

4**. Selection Criteria**
Inclusion criteria: Must include adaptation specifically for heat. All languages as long as an English abstract is available. Only reviews and original research articles as well as books or published national and international reports (defined as having an ISBN number). Must include at least one human health outcome. Must contain an evaluation or assessment. All publication years included.
Exclusion criteria: No English abstract available. Comments, editorials, correspondences and letters are excluded. Mitigation rather than adaptation focus of the article. Focus too limited: only a description of heat adaptation planned or implemented without assessment of effects. No evaluation of human health impacts. Purely economic analysis.

Two researchers will independently from each other select relevant articles from the searches with the same search terms as well as through cross-checking reference lists. One researcher will contact leading experts for input on work-in-progress and further studies to be included.
Disagreement between the two researchers will be resolved and evaluated by a third member of the research team.
Data extraction will occur for all studies included in the full-text reading phase.

5. Study Quality Assessment
Overall, this review adheres to the guidelines given in the PRISMA statement and checklists. For study quality assessment, the NHS Critical Appraisal Skills Programme (CASP) checklists will be used according to each study type. CASP also provides a checklist for quality appraisal of qualitative studies. Although specific tools for each study type prohibit a general comparison across study types, Katrak et al. (2005) have

previously criticized generic assessment tools for being too general. In addition, our review aims at being comprehensive and will therefore intentionally include a vast range of studies. Any attempt to assess these with a generic tool cannot represent their diversity. If an article does not fit a specific study type, we will assess the article guided by the following generic quality assessment questions (Booth et al. 2012):

1. Validity: Are the results of a study true? How are confounding and bias handled?
2. Reliability: What are the results and how much might they be due to chance?
3. Applicability: Can we generalize the results? How strong are recommendations for practice based on these study results?

Two reviewers will independently from each other assess study quality and then discuss their findings. Disagreements will be resolved by consultation with a third member of the research team. We will not weigh study quality by rating or scales: while study quality assessment is important to judge the overall evidence base for adaptation effectiveness, studies will not be excluded from the review based on their quality as exclusion of weaker studies has previously shown no effects in systematic literature reviews (Carroll et al. 2010; Thomas and Harden. 2008; Booth et al. 2012).

6. Data Extraction

We are using a data extraction form designed for the purposes of this review (see attachment). This data extraction form is based on the recommended form by Booth et al. (2012) but extended to include climate change adaptation specific questions (cf. Berrang-Ford et al. 2011). In addition, we made sure to consider all items required by the Cochrane Handbook for Systematic Reviews of Interventions (Higgins et al. 2005)
Data will be extracted into the data extraction form online (Word document) and added to an Excel spreadsheet for calculation/tabulation.
If applicable, effect sizes, ORs etc. will be taken directly from the studies. The data extraction form also offers space for free text and notes. Critical reflection by the researchers is encouraged.
Data extraction form attached.

7. Synthesis

Due to the expected heterogeneity and designs of studies and reports, no quantitative meta-analysis can be performed. Instead, synthesis will be achieved through narrative synthesis and tabulation of characteristics of adaptation measures, outcomes as well as study characteristics. (If studies are not as heterogeneous as currently expected, we will change synthesis type to meta-analysis.)
Threats to validity will be assessed by guiding questions on bias, mostly in the analysis phase.

8. Analysis

During the analysis phase we use tactics for testing or confirming review findings (Booth p. 171): subgroup analysis, identifying gaps, assessing shortcomings and limitations to evidence, identifying recommendations for action and future research. If we find extreme

outliers, we will consider sensitivity analyses by leaving out these studies on extreme ends of the spectrum. The analysis aims at providing quantitative and qualitative statements on effectiveness.

9. Study Limitations

Study type heterogeneity will likely prevent us from conducting a meta-analysis. Individually conducted evaluations of heat adaptation at regional level and evaluations by (non-)governmental organizations will not be included in this review unless they have been published as part of a national or international report. This makes our study vulnerable to publication bias.

10. Reporting

This review is part of one researcher's dissertation project. The results of this review will be published as an article with the target audience health researchers and climate change experts. Results will be reported according to the PRISMA statement guidelines.

11. Schedule

Task	Timescale	Dates
Meeting with supervisor to discuss Review Protocol	Month 0	Mid-October 2012<br>completed
Finalize scope	Month 0	October 2012<br>completed
Preliminary literature searches for clues/testing keywords	Month 0	October 2012<br>completed
Identify and contact key organizations (WHO, Tyndall, UNU EHS, UNEP, UNDP, WKC)	Month 1	January 2013<br>Moved to March 2013
Second meeting with supervisor:<br>Present finalized search protocol and terms	Month 1	January 2013<br>completed
	Month 1	January 2013<br>completed
Full literature searches and reference management with Mendeley	Months 2-3	March to April 2013
Selection of articles (read title and abstract)	Months 2-3	March to April 2013
Obtain articles from databases (full texts)	Months 2-3	March to April 2013
Follow-up cited references (hand search bibliographies)	Months 2-3	March to April 2013
Hand search extra journals	Months 2-3	March to April 2013

Meeting with supervisor	Month 3	April 2013
Appraisal: data extraction of full text articles	Month 3	April 2013
Data synthesis	Month 4	October 2013
Data analysis	Months 4-5	October 2013
Write results/prepare for publication	Month 5	November 2013
Correct and circulate report, write article	Months 5-6	November 2013
Prepare presentation of results for conference (EUPHA)	Months 5-6	EUPHA November 2013 2013

Appendix: Search strings and results

Searchstrings and hits

Pubmed

Search ((heat[Title/Abstract]) AND health[Title/Abstract]) AND evaluat*[Title/Abstract] Filters: Humans 310 Searched in: Pubmed
On: 07.03.2013

Search ((heat[Title/Abstract]) AND health[Title/Abstract]) AND assess*[Title/Abstract] Filters: Humans 333
Searched in: Pubmed
On: 07.03.2013

Search (((heat[Title/Abstract]) AND health[Title/Abstract]) AND adapt*[Title/Abstract]) AND (evaluat* OR assess*[Title/Abstract]) Filters: Humans 40
Searched in: Pubmed
On: 07.03.2013

Search (((heat[Title/Abstract]) AND health[Title/Abstract]) AND adapt*[Title/Abstract]) AND effectiv*[Title/Abstract] Filters: Humans 15
Searched in: Pubmed
On: 07.03.2013

Search (((heat[Title/Abstract]) AND health[Title/Abstract]) AND effectiv*[Title/Abstract]) AND (evaluat* OR assess*[Title/Abstract]) Filters: Humans 95
Searched in: Pubmed
On: 07.03.2013

Search ((heat exposure[Title/Abstract]) AND health[Title/Abstract]) AND (assess* OR evaluat*[Title/Abstract]) Filters: Humans 23
Searched in: Pubmed
On: 07.03.2013

(((heat exposure[Title/Abstract]) AND health[Title/Abstract]) AND adapt*[Title/Abstract]) AND effectiv*[Title/Abstract] Filters: Humans 2
Searched in: Pubmed
On: 07.03.2013

(((heat exposure[Title/Abstract]) AND health[Title/Abstract]) AND adapt*[Title/Abstract]) AND (evaluat* OR assess*[Title/Abstract]) Filters: Humans 1
Searched in: Pubmed
On: 07.03.2013

((heat island[Title/Abstract]) AND (evaluat* OR assess*[Title/Abstract])) AND effectiv*[Title/Abstract] Filters: Humans 2
Searched in: Pubmed
On: 07.03.2013

(heat stroke[Title/Abstract]) AND (evaluat* OR assess*[Title/Abstract]) Filters: Humans 85
Searched in: Pubmed
On: 07.03.2013

((heat stroke[Title/Abstract]) AND adapt*[Title/Abstract]) AND (evaluat* OR assess*[Title/Abstract]) Filters: Humans 1
Searched in: Pubmed
On: 07.03.2013

Search (((heat stroke[Title/Abstract]) AND adapt*[Title/Abstract]) AND (evaluat* OR assess*[Title/Abstract])) AND effectiv*[Title/Abstract] Schema: all Filters: Humans 0
Searched in: Pubmed
On: 07.03.2013

(((mortality[Title/Abstract]) AND heat[Title/Abstract]) AND adapt*[Title/Abstract]) AND (evaluat* OR assess*[Title/Abstract]) Filters: Humans 21
Searched in: Pubmed
On: 07.03.2013

Search (((mortality[Title/Abstract]) AND heat[Title/Abstract]) AND adapt*[Title/Abstract]) AND effectiv*[Title/Abstract] Filters: Humans 6
Searched in: Pubmed
On: 07.03.2013

Search ((climate change[Title/Abstract]) AND heat[Title/Abstract]) AND (evaluat* OR assess*[Title/Abstract]) Filters: Humans 49
Searched in: Pubmed
On: 07.03.2013

Search (((climate change[Title/Abstract]) AND heat[Title/Abstract]) AND adapt*[Title/Abstract]) AND effectiv*[Title/Abstract] Filters: Humans 9
Searched in: Pubmed
On: 07.03.2013

Search (((climate change[Title/Abstract]) AND heat[Title/Abstract]) AND adapt*[Title/Abstract]) AND (evaluat* OR assess*[Title/Abstract]) Filters: Humans 16

Searched in: Pubmed
On: 07.03.2013

=1008 hits

Web of Knowledge

Results
Title=(heat) AND Title=(health) AND Title=(evaluat*)
Timespan=All Years. Databases=SCI-EXPANDED, SSCI, A&HCI, CPCI-S, CPCI-SSH.

Results: 13

Title=(heat) AND Title=(health) AND Title=(assess*)
Timespan=All Years. Databases=SCI-EXPANDED, SSCI, A&HCI, CPCI-S, CPCI-SSH.

Results: 12

Heat AND health AND adapt* AND (evaluat* OR assess*) = 0

Heat AND health AND adapt* AND effectiv* = 0

Heat AND health AND effective* AND (evaluat* OR assess*)=0

Heat exposure AND health AND (evaluat* OR assess*)=0

Heat exposure AND health AND adapt* AND effective* =0

Heat exposure AND health AND (evaluat* OR assess*)=0

ResultsTitle=(heat island) AND Title=(evaluat* OR assess*) AND Title=(effectiv*)
Timespan=All Years. Databases=SCI-EXPANDED, SSCI, A&HCI, CPCI-S, CPCI-SSH.

Results: 1

ResultsTitle=(heat stroke) AND Title=(evaluat* OR assess*)
Timespan=All Years. Databases=SCI-EXPANDED, SSCI, A&HCI, CPCI-S, CPCI-SSH.

Results: 6

Heat stroke AND adapt* AND (evaluat* OR assess*) = 0

Heat stroke AND adapt* AND (evaluat* OR assess*) AND effective*= 0

Mortality AND heat AND adapt* AND (evaluat* OR assess*)=0

Mortality AND heat AND adapt* AND effectiv*=0

ResultsTitle=(climate change) AND Title=(heat) AND Title=(evaluat* OR assess*)
Timespan=All Years. Databases=SCI-EXPANDED, SSCI, A&HCI, CPCI-S, CPCI-SSH.

Results: 9

Climate change AND heat AND adapt* AND effectiv*=0

ResultsTitle=(climate change) AND Title=(heat) AND Title=(adapt*) AND
Title=(evaluat* OR assess*)
Timespan=All Years. Databases=SCI-EXPANDED, SSCI, A&HCI, CPCI-S, CPCI-SSH.

Results: 1

All hits= 42! (1 duplicate internal= 41 results)

PubMed MeSH with filter "Humans"

1. ((("Hot Temperature"[Mesh]) OR "Extreme Heat"[Mesh]) AND "Climate Change/mortality"[Mesh]) AND "Evaluation Studies as Topic"[Mesh]

No items found.

2. ((((("Hot Temperature"[Mesh]) OR "Extreme Heat"[Mesh]) AND "Climate Change/mortality"[Mesh]) AND "Health"[Mesh]) AND "Evaluation Studies"
No items found.

3. (("Hot Temperature"[Mesh] OR "Extreme Heat"[Mesh]) AND "Heat Stress Disorders"[Mesh]) AND ("Evaluation Studies as Topic"[Mesh] OR "Evaluation Studies" [Publication Type])

14 results.

4. (("Hot Temperature"[Mesh] OR "Extreme Heat"[Mesh]) AND "Health"[Mesh] AND("Risk Assessment"[Mesh] OR "Outcome and Process Assessment (Health Care)"[Mesh] OR "Health Impact Assessment"[Mesh])

18 results.

5. ((("Hot Temperature"[Mesh] OR "Extreme Heat"[Mesh]) AND "Heat Stress Disorders"[Mesh]) AND "Climate Change"[Mesh]) AND ("Risk Assessment"[Mesh] OR "Outcome and Process Assessment (Health Care)"[Mesh] OR "Health Impact Assessment"[Mesh])

4 results.

6. ("Hot Temperature"[Mesh] OR "Extreme Heat"[Mesh]) AND ("Evaluation Studies as Topic/methods"[Mesh] OR "Evaluation Studies as Topic/trends"[Mesh])

22 results.

7. (((("Hot Temperature"[Mesh]) OR "Extreme Heat"[Mesh]) AND "Climate Change/mortality"[Mesh]) AND "Risk Assessment"[Mesh]
No items found

8. ("Hot Temperature"[Mesh] OR "Extreme Heat"[Mesh]) AND "Risk Assessment"[Mesh]
158 results.

9. (("Climate Change"[Mesh]) AND ("Hot Temperature"[Mesh] OR "Extreme Heat"[Mesh])) AND "Risk Assessment"[Mesh]
11 results.

10. (("Heat Stress Disorders"[Mesh]) AND "Climate Change"[Mesh]) AND "Risk Assessment"[Mesh]
6 results.

11. (("Mortality"[Mesh]) AND "Climate Change"[Mesh]) AND ("Hot Temperature"[Mesh] OR "Extreme Heat"[Mesh])
32 results.

12. (("Mortality"[Mesh]) AND ("Hot Temperature"[Mesh] OR "Extreme Heat"[Mesh])) AND "Risk Assessment"[Mesh]
20 results.

13. (("Hot Temperature"[Mesh]) AND ("Heat Stress Disorders"[Mesh] OR "Heat Exhaustion"[Mesh] OR "Heat Stroke"[Mesh] OR "Extreme Heat"[Mesh])) AND "Evaluation Studies as Topic"[Mesh]
10 results.

Manual search:

Global Environmental Change:
http://www.sciencedirect.com/science/journal/09593780

Wiley Interdisciplinary Reviews: Climate Change:
http://wires.wiley.com/WileyCDA/WiresJournal/wisId-WCC.html

Nature und Nature Reports Climate Change:
http://www.nature.com/search/adv_search

Climatic Change:
http://link.springer.com/journal/10584/117/2/page/1

Int. Journal of Climate Change Strategies and Management:
http://www.emeraldinsight.com/journals.htm?issn=1756-8692)

heat AND adaptation AND evaluate
heat AND adaptation AND assess
heat AND adaptation AND effective
heat AND adaptation AND effect
heat stroke AND adaptation AND evaluate
heat stroke AND adaptation AND assess
heat stroke AND adaptation AND effective
heat stroke AND adaptation AND effect
mortality AND adaptation AND evaluate
mortality AND adaptation AND assess
mortality AND adaptation AND effective
mortality AND adaptation AND effect

UNFCCC library online:
https://unfccc.int/essential_background/library/items/3599.php

1. heat AND adaptation AND evaluate
0 results.
2. heat AND adaptation AND assess
2 results.
3. heat AND adaptation AND effective
3 results.
4. heat AND adaptation AND effect
7 results, all duplicates of the previous two searches.
5. heat stroke AND adaptation AND evaluate
0 results.
6. heat stroke AND adaptation AND assess
0 results.
7. heat stroke AND adaptation AND effective
0 results
8. heat stroke AND adaptation AND effect
0 results.
9. mortality AND adaptation AND evaluate
0 results.
10. mortality AND adaptation AND assess
1 result.
11. mortality AND adaptation AND effective
0 results.
12. mortality AND adaptation AND effect
1 result.

WHO digital library:
http://www.who.int/library/databases/en/

title search

1. heat AND adaptation AND evaluate
0 results.

2. heat AND adaptation AND assess
0 results.

3. heat AND adaptation AND effective
0 results.

4. heat AND adaptation AND effect
0 results.

5. heat stroke AND adaptation AND evaluate
0 results.

6. heat stroke AND adaptation AND assess
0 results.

7. heat stroke AND adaptation AND effective
0 results.

8. heat stroke AND adaptation AND effect
9. 0 results.

10. mortality AND adaptation AND evaluate

o results.
11. mortality AND adaptation AND assess
0 results.
12. mortality AND adaptation AND effective
0 results.
13. mortality AND adaptation AND effect
0 results.

Words/phrases search:
1 heat AND adaptation AND evaluate
0 results

2. heat AND adaptation AND assessment
2 results.

3. heat AND adaptation AND effective
0 results
4. heat AND adaptation AND effect
O results

5. heat stroke AND adaptation AND evaluate
0 results.

6. heat stroke AND adaptation AND assess
0 results.

7. heat stroke AND adaptation AND effective
0 results.

8. heat stroke AND adaptation AND effect
0 results.

9. mortality AND adaptation AND evaluate
0 results.

10.mortality AND adaptation AND assess
0 results, with assessment 1 result that is a duplicate from no. 2
11.mortality AND adaptation AND effective
0 results
12. mortality AND adaptation AND effect
0 results

Global Environmental Change:
http://www.sciencedirect.com/science/journal/09593780

1. heat AND adaptation AND evaluate
61 results.

2. heat AND adaptation AND assess
111 results.

3. heat AND adaptation AND effective
104 results.

4. heat AND adaptation AND effect
159 results.

5. heat stroke AND adaptation AND evaluate
1 result.

6. heat stroke AND adaptation AND assess

5 results.

7. heat stroke AND adaptation AND effective
5 results.

8. heat stroke AND adaptation AND effect
7 results.

9. mortality AND adaptation AND evaluate
30 results.

10. mortality AND adaptation AND assess
63 results.

11. mortality AND adaptation AND effective
73 results.

12. mortality AND adaptation AND effect

100 results.

Wiley Interdisciplinary Reviews: Climate Change: http://wires.wiley.com/WileyCDA/WiresJournal/wisId-WCC.html

1. heat AND adaptation AND evaluate
0 results

2. heat AND adaptation AND assess
0 results

3. heat AND adaptation AND effective
0 results
4. heat AND adaptation AND effect
1 result.

5. heat stroke AND adaptation AND evaluate
0 results

6. heat stroke AND adaptation AND assess
0 results

7. heat stroke AND adaptation AND effective
0 results

8. heat stroke AND adaptation AND effect
0 results

9. mortality AND adaptation AND evaluate
0 results.
10. mortality AND adaptation AND assess
0 results.

11. mortality AND adaptation AND effective
0 results.

12. mortality AND adaptation AND effect

0 results.

Nature Climate Change und Nature Reports Climate Change: http://www.nature.com/search/adv_search

- use without AND as one search string indicating „ all words “ which means the same thing!
- Apply filter article type: research

1. heat adaptation evaluation
34 hits

2. heat adaptation assessment
9 results.

3. heat adaptation effective

34 results.

4. heat adaptation effect

9 results.

5. heat stroke adaptation evaluation
0 results.

6. heat stroke adaptation assessment
0 results.

7. heat stroke adaptation effective
0 results.

8. heat stroke adaptation effect
0 results.

9. mortality adaptation evaluation
1 result.

10. mortality adaptation assessment
0 results.
11. mortality adaptation effective
1 result.
12. mortality adaptation effect
1 result.

Climatic Change:
http://link.springer.com/journal/10584/117/2/page/1

1. heat AND adaptation AND evaluate
299 results.

2. heat AND adaptation AND assess
402 results

3. heat AND adaptation AND effective
242 results.

4. heat AND adaptation AND effect

467 results.

5. heat stroke AND adaptation AND evaluate

6 results.

6. heat stroke AND adaptation AND assess
6 results.

7. heat stroke AND adaptation AND effective
2 results.

8. heat stroke AND adaptation AND effect
6 results.

9. mortality AND adaptation AND evaluate
106 results.

10. mortality AND adaptation AND assess
158 results.

11. mortality AND adaptation AND effective
97 results.

12. mortality AND adaptation AND effect

184 results.

Int. Journal of Climate Change Strategies and Management: http://www.emeraldinsight.com/journals.htm?issn=1756-8692

1. heat AND adaptation AND evaluate
9 results.

2. heat AND adaptation AND assess
14 results.

3. heat AND adaptation AND effective
16 results.

4. heat AND adaptation AND effect
18 results.

5. heat stroke AND adaptation AND evaluate
38 results.

6. heat stroke AND adaptation AND assess
38 results (likely duplicates)

7. heat stroke AND adaptation AND effective
38 results.

8. heat stroke AND adaptation AND effect
38 results.

9. mortality AND adaptation AND evaluate
98 results.

10. mortality AND adaptation AND assess
5 results.

11. mortality AND adaptation AND effective
6 results.

12. mortality AND adaptation AND effect
9 results.
