# Supplementary material for: Is planned adaptation to heat reducing heat-related mortality and illness? A systematic review
Source: BMC Public Health. 2014 Oct 28;14:1112. doi: 10.1186/1471-2458-14-1112 (PMC4219109; doi:10.1186/1471-2458-14-1112)
Supplement: Supplementary file 3 — Additional file 3: Data extraction sheet. (PDF 209 KB) [file 12889_2014_7199_MOESM3_ESM.pdf]

<u>Data Extraction Form</u>
<u>For all texts in the full-text reading phase</u>

Title of Review: Is adaptation to heat reducing heat-related mortality and illness? A systematic review

Review Reference no.

1. Publication Details

1.1 *Author(s)*:

1.2. *Country of authors (First author if several):*

1.3.*Year of publication:*

1.4 *Reviewer comments:*

1.5 *Eligible for review?*

1.6 *If excluded, reasons for exclusion:*

1.7 *Authors' conclusions (short summary):*

2. Study details

2.1 *Study aims* (main research question):

2.2 *Additional research questions addressed*:

2.3 *Study design*:

Quantitative: qualitative: mixed methods:

Other study type:

2.4 *Study type:*

2.5 *Methods of data collection:*

2.6 *Who collected data?*

Researchers:

Peers (trained participants, participatory approach):

Secondary data set used:

Other person:

2.7 *Methods used:*

2.8 Conceptual/theoretical framework underlying the study:

2.9 *Country or Countries in which study was conducted*

2.10 *Study duration:*

2.11 *Any stakeholders involved in study design or conduct?*

2.12 [Target population (if applicable):

Participant number

age,

0-20 years:

21-60:

60plus:

gender,

ethnicities,

socio-demographic information,

Morbidities

Sampling strategy for participant recruitment

2.13 Which **health outcomes** are studied?

2.14 Which **other effects** are studied if any?

3. Adaptation-specific general information:
3.1 *Time frame of*

a) Adaptation measure:

b) Effects :

3.2 *Spatial scale of adaptation*:

Community level (i.e .one city, or one part of a city):

Regional level:

National level:

Global level (an adaptation program designed to be implemented in several countries at the same time with the same design):

No info available:

3.3 *Region of the adaptation measure*:

North America:

Central America:

South America:

Western Europe:

Central Europe:

Eastern Europe:

Middle East:

Northern Africa:

Sub-Saharan Africa:

Central and West Asia:

East Asia:

South-East Asia and Pacific:

South Asia:

Australia & New Zealand:

3.4 *Sectors studied*:
Health sector:

Other sectors (please specify):

3.5 *Stakeholders involved in adaptation*:

If none:

NGO municipal:

NGO national:

NGO international:

Government municipal:

Government national:

Government international (i.e. UN):

Private industry:

Households or individuals:

3.6 Vulnerable groups specifically mentioned as target population?

Coastal cities:

Small island states:

Women:

Children:

Older persons:

Lower income populations:

Other (please specify):

4. Adaptation process and outcomes:
4.1 Timing:

Anticipatory:

Responsive:

No information available:

4.2 Type of adaptation:

Structural (infrastructural, i.e. dams):
Behavioral (includes awareness raising, perception):

Warning system:

Technological adaptation EXCEPT warning system (i.e. air conditioning):

Emergency action plan (includes all emergency policy plans):

4.3 *Evaluation of adaptation conducted?*

Yes: no:

4.4 *Outcome measures used:*

4.5 *Details of findings:*

Effects estimated? Effect sizes mentioned?

Yes: No: n/a:

If yes: extract exact numbers for further calculations.

(Reminder:
Outcomes*: impacts on heat stroke incidence and cardiovascular mortality. Impacts measured as reduction in heat stroke incidence and cases of cardiovascular mortality in extreme heat periods.*
Effectiveness measured medically as reduction in heat stroke incidence and cardiovascular mortality. Effectiveness measured by heat island exposure reduction signaled through changes in these risk factors:

- *awareness of heat-appropriate behavior,*
- *green roofs,*
- *air conditioning,*
- *shaded buildings,*
- *early heat wave warning systems,*
- *risk communication strategies.)*
