# Supplementary material for: Is planned adaptation to heat reducing heat-related mortality and illness? A systematic review
Source: BMC Public Health. 2014 Oct 28;14:1112. doi: 10.1186/1471-2458-14-1112 (PMC4219109; doi:10.1186/1471-2458-14-1112)
Supplement: Supplementary file 4 — Additional file 4: Quality appraisal results. The results of the quality appraisal conducted with CASP checklists. (DOCX 24 KB) [file 12889_2014_7199_MOESM4_ESM.docx]

**Supplementary Table 1: QUALITY ASSESSMENT RESULTS**

All questions © CASP

**Legend: Y= Yes, N=No, C= Cannot tell**

CASP CHECKLIST FOR CASE-CONTROL STUDIES (selected questions applied to regression analysis studies)

| **Reference** | **Did the study address a clearly focused issue?** | **Did the authors use an appropriate method to answer the question?** | **Were the cases recruited in an acceptable way?** | **Were the controls selected in an acceptable way?** | **Was the exposure accurately measured to minimize bias?** | **Taken account of confounding?** | **Discussed how precise the results are (CI, p-values given)?** | **Do the results of the study fit with other available evidence?** | **Comments** |
| --- | --- | --- | --- | --- | --- | --- | --- | --- | --- |
| Chau et al. 2009 | Y | Y | Y | C | Y | Y | Y | Y | Case-only study |
| Davis et al. 2003 | Y | Y | Y | C | Y | Y | N | Y |  |
| De’Donato et al. 2008 (only abstract available) | Y | Y | C | C | Y | Y | C | Y |  |
| Delaroziere & Sanmarco 2004 | Y | Y | Y | C | Y | C | Y | Y |  |
| Fouillet et al. 2008 | Y | Y | Y | C | Y | Y | Y | Y | Control= expected excess mortality |
| Kysely & Kriz 2008 | Y | Y | Y | C | Y | Y | Y | Y | Control= period in time |
| Kysely & Plavcova 2012 | Y | Y | Y | C | Y | Y | Y | Y |  |
| Mattern et al. 2000 | Y | Y | Y | C | C | N | N | Y | Exposure= knowledge changes |
| Morabito et al. 2012 | Y | Y | Y | C | Y | Y | Y | Y |  |
| Ostro et al. 2010 | Y | Y | Y | Y | C | Y | Y | Y |  |
| Palecki et al. 2001 | Y | Y | C | C | C | N | N | C | No conclusive results given |
| Schifano et al. 2012 | Y | Y | Y | Y | Y | Y | Y | Y | Days with low temperature are controls |
| Smoyer 1998 | Y | Y | Y | C | Y | Y | Y | N | Reported increased mortality whereas most studies report downward mortality trends |
| Tan et al. 2007 | Y | Y | C | C | Y | Y | Y | Y | No information on source of mortality data |
| Weisskopf et al. 2002 | Y | Y | Y | n.a. | Y | N | Y | Y | Study projects deaths only |

CASP CHECKLIST FOR COHORT STUDIES

| **Reference** | **Did the study address a clearly focused issue?** | **Was the cohort recruited in an acceptable way?** | **Was the exposure accurately measured?** | **Was the outcome accurately measured?** | **Taken account of confounding?** | **Follow-up of studies complete enough?** | **Discussed how precise the results are (CI, p-values given)?** | **Do the results of the study fit with other available evidence?** | **Comments** |
| --- | --- | --- | --- | --- | --- | --- | --- | --- | --- |
| Bargagli et al. 2009 | Y | Y | Y | Y | C | Y | Y | C | No similar studies of GP programs so far |
| Rogot et al. 1992 | Y | Y | Y | Y | C | Y | Y | Y | Exposure= air conditioning prevalence |

CASP CHECKLIST FOR RCT

| **Reference** | **Did the trial address a clearly focused question?** | **Was the assignment of patients to treatments randomized?** | **Were all patients who entered the trial properlyaccounted for at its end?** | **Was the trial blind?** | **Were the groups similar at the start of the trial?** | **Discussed how precise the results are (CI, p-values given)?** | **Can the results be applied in the local population?** | **Comments** |
| --- | --- | --- | --- | --- | --- | --- | --- | --- |
| Marinacci et al. 2009 | Y | Y | C | N | C | Y | Y | Trial could not be blind as it was an intervention |

CASP CHECKLIST FOR QUALITATIVE RESEARCH (applied also to quantitative survey research)

| **Reference** | **Was there a clear statement of the aims of the research?** | **Is the methodology appropriate?** | **Was the research design appropriate?** | **Was the recruitment strategy appropriate?** | **Was the data collected in a way that addressed the research issue?** | **Have ethical issues been considered?** | **Was the data analysis rigorous?** | **Is there a clear statement of findings?** | **Comments** |
| --- | --- | --- | --- | --- | --- | --- | --- | --- | --- |
| Abrahamson et al. 2009 | Y | Y | Y | Y | Y | C | Y | Y | Qualitative interviews |
| Bittner & Stößel 2012 | Y | Y | Y | Y | Y | N | Y | Y | Qualitative interviews |
| Kalkstein & Sheridan 2007 | Y | Y | Y | N | Y | N | Y | Y | Quantitative, did not reach most vulnerable group |
| Kishonti et al. 2006 (only abstract) | Y | N (no baseline) | N (no baseline) | Y | Y | N | C | Y | Quantitative |
| Kosatsky et al. 2009 | Y | Y | Y | Y | Y | Y | Y | Y | Quantitative |
| Oakman et al. 2010 | Y | Y | Y | Y | Y | N | Y | Y | Oakman et al. 2010 |
| Sheridan 2007 | Y | Y | Y | Y | Y | N | Y | Y | Quantitative |

CASP CHECKLIST FOR ECONOMIC EVALUATION

| **Reference** | **Was a well-defined question posed?** | **Was a comprehensive description of competing alternatives given?** | **Does the paper provide evidence that the program would be effective?** | **Were the effects of the intervention measured and valued appropriately?** | **Were all important and relevant resources required and health outcome costs identified, measured and valued appropriately?** | **Discounting?** | **Sensitivity analysis?** |
| --- | --- | --- | --- | --- | --- | --- | --- |
| Ebi et al. 2004 | Y | C | C | Y | Y | Y | N |

CASP CHECKLIST FOR STRUCTURED REVIEWS

| **Reference** | **Did the review address a clearly focused question?** | **Did the authors look for the right type of papers?** | **Do you think important studies were included?** | **Sufficient quality assessment?** | **If results were combined, reasonable to do so?** | **Discussed how precise the results are (CI, p-values given)?** | **All important outcomes considered?** | **Comments** |
| --- | --- | --- | --- | --- | --- | --- | --- | --- |
| Bassil & Cole 2010 | Y | Y | Y | C | n.a. | N | Y | Narrative synthesis |
| Bouchama et al. 2007 | Y | Y | Y | Y | N | Y | Y | Combined very heterogeneous studies for meta-analysis |
| Gupta et al. 2012 | Y | Y | n.a. | n.a. | n.a. | n.a. | Y | Did not include any studies as none qualified |
| Toloo et al. 2013 | Y | C | C | N | n.a. | N | Y | No details given on selection criteria, search strategy or quality appraisal |
