# Supplementary material for: Is planned adaptation to heat reducing heat-related mortality and illness? A systematic review
Source: BMC Public Health. 2014 Oct 28;14:1112. doi: 10.1186/1471-2458-14-1112 (PMC4219109; doi:10.1186/1471-2458-14-1112)
Supplement: Supplementary file 5 — Additional file 5: Table of characteristics of studies included in review. Studies are presented ordered by type and year of publication. (DOCX 21 KB) [file 12889_2014_7199_MOESM5_ESM.docx]

**Additional file 5: Characteristics of studies included in the review**

| ***Study type*** | ***Reference*** | ***Year*** | ***City, Country of study*** | ***Study population characteristics*** |
| --- | --- | --- | --- | --- |
| RCT | Marinacci et al. 2006 | 2006 | Turin, Italy | 75years+, living alone, clinically and/or functionally diagnosed as frail |
|  |  |  |  |  |
| Systematic Review | Bouchama et al. 2007 | 2007 | not restricted | Whole population |
|  | Bassil & Cole 2010 | 2010 | not restricted | Whole population |
|  | Gupta et al. 2012 | 2012 | not restricted | Whole population in low-, middle- and high-income countries, focus on  vulnerable groups planned |
|  | Toloo et al. 2013 | 2013 | not restricted | Whole population |
|  |  |  |  |  |
| Qualitative interview study | Abrahamson et al. 2009 | 2009 | London and Norwich, UK | 72 to 94 years old, living in their own homes in urban areas in Norwich and London.  From the 20% most deprived and 20% least deprived areas based on a national index |
|  | Bittner & Stößel 2012 | 2012 | Freiburg, Germany | 64 to 94 years, living alone or in nursing homes |
|  |  |  |  |  |
| Quantitative survey research | Mattern et al. 2000 | 2000 | North Philadelphia, Penn., USA | Entire population of North Philadelphia, of which 41.9% to 49.6% of respondents living below poverty level |
|  | Kishonti et al. 2006 | 2006 | 5 towns in Hungary | Over 18 years old, have telephone |
|  | Kalkstein & Sheridan 2007 | 2007 | Phoenix, Arizona, USA | Entire population of Phoenix meteorological area, sampling in front  of shopping centers |
|  | Sheridan 2007 | 2007 | Dayton(Ohio, USA), Philadelphia (Pennsylvania, USA), Phoenix (Arizona, USA), and Toronto (Ontario, Canada) | 65 years +, living at home |
|  | Kosatsky et al. 2009 | 2009 | Montreal, Quebec, Canada | Clinic patients with heart failure or COPD, residing at home in  Montreal and with telephone access and spoke English or French |
|  | Oakman et al. 2010 | 2010 | Riverina-Murray Region, New South Wales, Australia | Over 18 years old, have telephone |
|  |  |  |  |  |
| Multivariate analysis or time series study | Smoyer 1998 | 1998 | St. Louis, Missouri | 64 years + in St. Louis, Missouri |
|  | Palecki et al. 2001 | 2001 | Midwest USA (Illinois, Indiana, Iowa, Kentucky, Michigan, Minnesota, Missouri, Ohio, Wisconsin) | Entire population of Chicago and St Louis |
|  | Weisskopf et al. 2002 | 2002 | Milwaukee, Wisconsin, USA | Entire population of Milwaukee, WS |
|  | Davis et al. 2003 | 2003 | 28 cities in the USA | Entire population of the 28 cities in the US |
|  | Delaroziere & Sanmarco 2004 | 2004 | Marseille, France | 65 years+ in Marseille |
|  | Tan et al. 2007 | 2007 | Shanghai, China | Entire population of Shanghai, China |
|  | De'Donato et al. 2008 | 2008 | 17 cities in Italy | Population of 17 cities in Italy, no further information |
|  | Fouillet et al. 2008 | 2008 | France | Entire population of France from 1975 to 2006 |
|  | Kysely & Kriz 2008 | 2008 | Czech Republic | Entire population of CZ from 1986 to 2006 |
|  | Chau et al. 2009 | 2009 | Hong Kong | 65 years + in Hong Kong |
|  | Ostro et al. 2010 | 2010 | California, USA | Entire population of California: hospital admissions between 1999 and 2005 for  cardiovascular and respiratory diseases, diabetes, dehydration, heat stroke,  intestinal infection, acute renal failure |
|  | Kysely & Plavcova 2012 | 2012 | Czech Republic | Entire population of CZ from 1986 to 2009 |
|  | Morabito et al. 2012 | 2012 | Florence, Italy | 65 to 74 years of age and 75 years+ |
|  | Schifano et al. 2012 | 2012 | 16 cities in Italy | 65 years +, living in the 16 cities |
|  |  |  |  |  |
| Economic evaluation | Ebi et al. 2004 | 2004 | Philadelphia, Pennsylvania, USA | 65 years+ in Philadelphia |
|  |  |  |  |  |
| Cohort study | Rogot et al. 1992 | 1992 | USA | Study population of National Longitudinal Mortality Study, 2 cohort samples.  72.740 persons |
|  | Bargagli et al. 2009 | 2009 | Rome, Italy | 65 years +, preexisting conditions, regularly seeing GP |
